# Exogenously Induced Silencing of Four MYB Transcription Repressor Genes and Activation of Anthocyanin Accumulation in *Solanum lycopersicum*

**DOI:** 10.3390/ijms24119344

**Published:** 2023-05-26

**Authors:** Andrey R. Suprun, Konstantin V. Kiselev, Alexandra S. Dubrovina

**Affiliations:** Laboratory of Biotechnology, Federal Scientific Center of the East Asia Terrestrial Biodiversity, Far Eastern Branch of the Russian Academy of Sciences, Vladivostok 690022, Russia

**Keywords:** anthocyanins, double-stranded RNAs, RNA interference, tomato, transcription factors

## Abstract

RNA interference (RNAi) is a natural post-transcriptional regulatory mechanism that can be artificially induced by exogenous application of double-stranded RNAs (dsRNAs) to the plant surfaces. Recent studies show that it is possible to silence plant genes and change plant properties using plant RNA spraying and other approaches for dsRNA delivery. In this study, we investigated the effect of exogenous gene-specific dsRNAs on the silencing of four tomato genes encoding MYB-family transcription repressors of anthocyanin biosynthesis in the leaves of tomato *Solanum lycopersicum* L. We found that the exogenous application of dsRNAs encoding for the *SlMYBATV1*, *SlMYB32*, *SlMYB76*, and *SlTRY* genes downregulated mRNA levels of these endogenous repressors of anthocyanin production, upregulated the expression of anthocyanin biosynthesis-related genes, and enhanced anthocyanin content in the leaves of *S. lycopersicum*. The data demonstrated that exogenous gene-specific dsRNAs can induce post-transcriptional gene silencing in tomato leaves by direct foliar application of dsRNAs. This approach may be used for plant secondary metabolism induction and as a silencing tool for gene function studies without the need to produce genetically modified plants.

## 1. Introduction

RNA interference (RNAi) is a process of post-transcriptional suppression (or silencing) of gene expression in plants and other eukaryotic organisms where small non-coding RNAs of 20–25 nucleotides function as key players and are generated from longer double-stranded RNAs (dsRNAs) [1,2]. The dsRNAs may enter plant vascular systems exogenously as plant dsRNA viruses or are formed endogenously after the transcription of certain sections in the plant genome. The dsRNA precursors are processed by specialized ribonuclease III-like enzymes (DICER LIKE or DCL) into small interfering RNAs (siRNAs), which are then incorporated into the RNA-induced protein complex (RISC) [1,3]. RISC destroys any mRNA molecules similar to the introduced dsRNAs. It is known that the RNAi phenomenon is implicated in the regulation of various processes in plants, such as in plant defense from pathogenic viruses and microorganisms, plant growth and development, abiotic stress adaptation, or the synthesis of secondary metabolites [1,4,5]. 

Currently, the phenomenon of RNAi is widely exploited as a powerful tool in experimental plant biology for gene silencing, in gene functional studies, and for engineering of valuable crop traits [6,7,8,9]. The major RNAi-based strategies for crop improvement and plant protection include host-induced gene silencing (HIGS) [10], virus-induced gene silencing (VIGS) [11], insect pest management by feeding insects with dsRNA [12], and external dsRNA application, termed as spray-induced gene silencing (SIGS) or exogenous RNAi (exo-RNAi) [13,14].

Recent studies show that exogenously applied dsRNAs (for example, by simple spraying, spraying under high pressure or with the application of nanocarriers) are able to penetrate into the plant vascular system and are delivered into plant cells [15,16,17,18,19]. Then, the dsRNAs are processed into siRNAs and induce the RNAi-mediated silencing of genes essential in attacking plant pathogens and plant viruses, leading to the alleviation of the negative effects associated with the plant pathogen attack and plant resistance induction [16,17,20,21,22,23,24,25,26]. Plant exogenous treatment with gene-specific dsRNAs or siRNAs followed by silencing of a target gene is currently termed as spray-induced gene silencing (SIGS) or exogenously induced plant RNAi (exo-RNAi), and is considered as an attractive strategy for crop improvement and gene functional studies [12,27,28]. While there is a considerable number of studies reporting on the dsRNA-induced silencing of virulence genes in attacking plant microbial pathogens or dsRNA antiviral effects [8,9,12,29], much less is known about exogenously induced gene silencing in the plant genome itself. Several studies [30,31,32,33] and one patent [34] reported on the silencing of plant genes after the exogenous application of dsRNAs/siRNAs. These investigations showed that external plant treatments with dsRNAs encoding for target genes in the plant genome downregulated the expression of the gene targets, i.e., the 3-phosphate synthase (*EPSPS*) gene in tobacco and amaranth leaves [34], the *Myb1* gene in orchid flower buds [30], the *Mob1A*, *WRKY23*, and *Actin* genes in *Arabidopsis thaliana* [31], and the *LBDIf7* and *GST40* genes in grapevine [32,33]. According to the studies, external treatment of the plant surfaces with the gene-specific dsRNAs led to expected phenotypic or biochemical changes, such as changes in the morphology of flowers [30], improved plant resistance to fungal infection [32], and improved drought stress tolerance [33]. In addition, there have been reports where the silencing of plant target genes was achieved using nanoparticle-mediated [35,36] or laser light-accompanied [37] plant exogenous treatments with dsRNAs. Overall, the number of studies is limited, and the review of current research [13,14] shows that further studies are needed for the development of simple and safe approaches for RNAi induction and the target-specific downregulation of plant genes. Much remains unknown about the possibility and effectiveness of plant gene regulation by exogenous dsRNAs, especially for agricultural crops.

Recently, we have found that the foliar treatments of *A. thaliana* with dsRNAs and siRNAs targeting the chalcone synthase (*AtCHS*) gene, encoding for a key enzyme in anthocyanin biosynthesis, or *AtMybL2* and *AtANAC032* genes of two transcription repressors of anthocyanin biosynthesis, have led to an RNAi-mediated decrease in the content of the corresponding mRNA transcripts [19,38]. This gene silencing effect resulted in alterations in the content of anthocyanins, including a reduction in anthocyanin level after application of *AtCHS*-dsRNA and the enhancement of anthocyanin levels after application of *AtMybL2*-dsRNA or *AtANAC032*-dsRNA [19,38]. Anthocyanins are blue, red, or purple plant pigments derived from the phenylpropanoid pathway that provide coloration to flowers, fruits, and vegetables and attract pollinators/seed distributors and protect plants from a variety of environmental stresses [39,40,41]. Anthocyanins also play an important role in plant stress protection [42] and possess health-promoting antioxidant effects used in medicine, cosmetology, and food industry [39]. While anthocyanins are produced by many plant species, they are absent in most cultivated tomatoes due to the incomplete activation of the flavonoid biosynthetic pathway [43,44]. Tomato is an attractive plant species for application of the new SIGS technology, for both the development of new biotechnological approaches for activation of anthocyanin biosynthesis and as a model plant for plant gene functional studies. 

In this study, we aimed to activate anthocyanin levels in tomato *Solanum lycopersicum* L. by plant spraying with dsRNAs targeting several MYB-family repressors of anthocyanin biosynthesis. Currently, it is established that the biosynthesis of anthocyanins is controlled by the MYB-bHLH-WD40 (MBW) transcriptional complex, composed of three classes of transcriptional regulators, including the MYB transcription factors, basic helix-loop-helix (bHLH), and WD-repeat (WDR) proteins [45]. MYB-family transcription factors determine the activator or repressor role of the MBW complex by binding to the promoters of structural biosynthetic genes in the complex with the bHLH and WD40 factors. In addition, a variety of additional transcriptional regulators have been discovered that destabilize the MBW complex and counteract with its transcriptional activity, decreasing anthocyanin production [45]. SlMYBATV (*S. lycopersicum* MYB at the atroviolacium locus), a R3-type MYB transcription factor, is the most studied MYB-family transcriptional repressor of anthocyanin biosynthesis in tomato [46,47]. According to Cao et al. 2017 [46], tomato plant lines harboring the *SlMYBATV* gene mutation exhibited increased expression of the structural and regulatory genes of anthocyanin biosynthesis. Colanero et al. 2018 [47] provided further evidence for the role of SlMYBATV as a repressor of anthocyanin biosynthesis demonstrating that overexpression of *SlMYBATV* reduced anthocyanin production. Colanero et al. 2018 [47] found that the SlMYBATV interacts with bHLH tomato factors involved in the MBW complexes, which leads to disruption of its activity and repressed anthocyanin production. Two other MYB-family regulators have been identified as putative R3-type MYB repressors (SlTRY and SlMYBATV-like) and four as R2R3-type MYB repressors (SlMYB3, SlMYB7, SlMYB32, and SlMYB76) of anthocyanin biosynthesis in tomato using a genome-wide screen [48]. A study by Nukumizu et al. 2013 [49] supported the repressor role of SlTRY in anthocyanin biosynthesis, since *SlTRY* overexpression in Arabidopsis reduced anthocyanin accumulation. To the best of our knowledge, further conclusive information on anthocyanin biosynthesis regulation by the putative regulators is still lacking. Therefore, *SlMYBATV*, as the best-studied repressor, and three other putative repressor genes, including *SlMYB32*, *SlMYB76*, and *SlTRY,* were selected as target genes in the present work.

In the present study, we aimed to evaluate the effect of tomato spraying with gene-specific dsRNAs on the mRNA levels of the four selected MYB-family transcriptional repressors of anthocyanin biosynthesis, including *SlMYBATV*, *SlMYB32*, *SlMYB76*, and *SlTRY* genes, and on the mRNA levels of some anthocyanin biosynthesis genes, including two chalcone synthase genes (*SlCHS1*, *SlCHS2*) and an anthocyanin synthase gene (*SlANS*). Then, we studied the effect of the gene-specific dsRNAs on anthocyanin contents and profile.

## 2. Results

### 2.1. Exogenous dsRNAs Downregulate mRNA Levels of SlMYBATV, SlMYB32, SlMYB76, and SlTRY Transcription Factors

**Figure 1:**
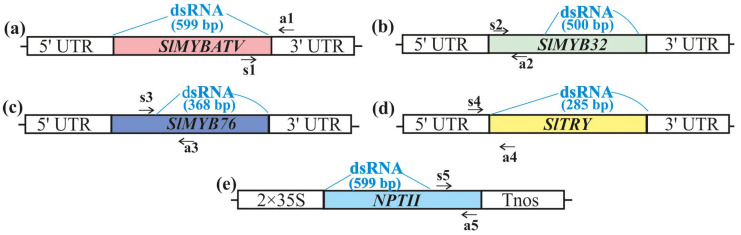
Schematic representation of the dsRNAs and the qRT–PCR primer positions designed to verify the effects of external dsRNA treatments on the levels of endogenous *SlMYBATV*, *SlMYB32*, *SlMYB76*, and *SlTRY* mRNAs. (**a**) Representation of *SlMYBATV* cDNA coding region with positions of the *SlMYBATV*-dsRNA and primers; (**b**) representation of *SlMYB32* cDNA coding region and position of *SlMYB32*-specific dsRNA and primers; (**c**) representation of *SlMYB76* cDNA coding region and position of *SlMYB76*-dsRNA and primers; (**d**) representation of *SlTRY* cDNA coding region with position of the *SlTRY*-dsRNA and primers; (**e**) representation of *NPTII* coding region and positions of the *NPTII*-specific dsRNA and primers. Black arrows indicate positions of the primers (s1, a1, s2, a2, s3, a3, s4, a4, s5, a5) used for *SlMYBATV*, *SlMYB32*, *SlMYB76*, and *SlTRY* mRNA analysis. UTR—untranslated region, 2 × 35S—the double 35S promoter of the cauliflower mosaic virus (CaMV), Tnos—nopaline synthase terminator.

We used PCR and an in vitro transcription protocol to produce dsRNAs of the *SlMYBATV*, *SlMYB32*, *SlMYB76*, and *SlTRY* genes (Figure 1), encoding transcriptional repressors and negatively regulating anthocyanin biosynthesis in *S. lycopersicum* [46]. We also obtained dsRNA targeting a non-related bacterial neomycin phosphotransferase II (*NPTII*) gene (Figure 1), to show whether any observed dsRNA-related effects are sequence-specific.

Full-length coding cDNAs of the *SlMYBATV*, *SlMYB32*, *SlMYB76*, *SlTRY*, and *NPTII* genes were amplified (Figure 1). The obtained PCR products, containing T7 promoters at both ends, were used as templates for in vitro transcription. For external plant treatments, 70 µg of the synthesized dsRNAs were diluted in water to a final concentration of 0.175 µg/µL and applied on the foliar surface of an individual *S. lycopersicum* plant by spraying. Four-week-old *S. lycopersicum* was treated at a late day time (21:00–21:30) under low soil moisture conditions, since these parameters were important for successful gene silencing in transgenic *A. thaliana* according to our analysis [19,50,51].

**Figure 2:**
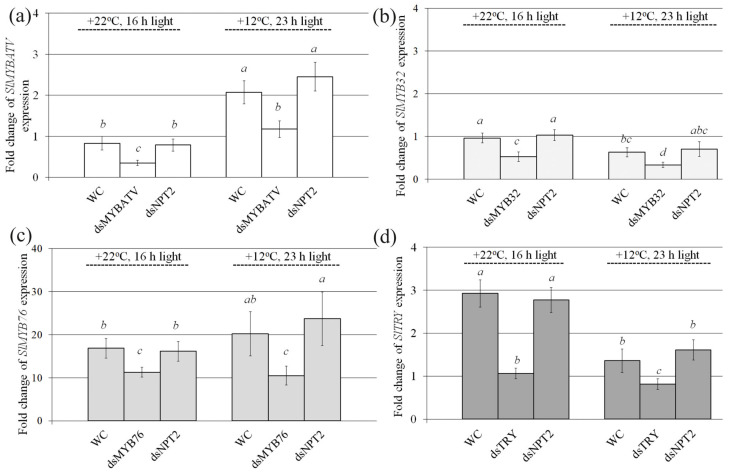
Relative fold change in *SlMYBATV* (**a**), *SlMYB32* (**b**), *SlMYB76* (**c**), and *SlTRY* (**d**) mRNA levels after dsRNA treatments of *Solanum lycopersicum* compared to untreated plants. WC—*S. lycopersicum* treated with sterile water; dsMYBATV—*S. lycopersicum* treated with *MYBATV*-dsRNA; dsMYB32—*S. lycopersicum* treated with *SlMYB32*-dsRNAs; dsMYB76—*S. lycopersicum* treated with *SlMYB76*-dsRNAs; dsTRY—*S. lycopersicum* treated with *SlTRY*-dsRNAs; dsNPT2—*S. lycopersicum* treated with *NPTII*-dsRNA. Total RNA was isolated seven days after dsRNA application and quantitative real-time PCR was used for gene expression analysis. *S. lycopersicum* was grown under control (+22 °C, 16 h light) and anthocyanin-inducing (+12 °C, 23 h light) conditions. The data are presented as the mean ± SE (three independent experiments). Means on each figure followed by the same letter were not different using one-way analysis of variance (ANOVA), followed by the Tukey HSD multiple comparison test.

Then, we analyzed whether exogenous application of the pure *SlMYBATV*-, *SlMyb32*-, *SlMYB76*-, *SlTRY*-, and *NPTII*-dsRNAs to the foliar surface of four-week-old *S. lycopersicum* could lead to any changes in the mRNA transcript levels of the *SlMYBATV*, *SlMYB32*, *SlMYB76*, and *SlTRY* genes in comparison with the water-treated controls seven days after dsRNA application (Figure 2). Since under standard cultivation conditions, anthocyanin production and the expression of anthocyanin biosynthesis genes in *S. lycopersicum* were low, we divided the treated *S. lycopersicum* into two groups for post-treatment incubation–plants cultivated under control conditions (+22 °C, 16 h light) or anthocyanin-inducing conditions (+12 °C, 23 h light)—for seven days in order to induce *SlCHS* expression and anthocyanin biosynthesis and analyze the dsRNA effects.

The qRT–PCR analysis revealed that the mRNA level of the *SlMYBATV* gene was considerably lower after plant foliar treatment with *SlMYBATV*-dsRNA than after the application of water or the nonspecific *NPTII*-dsRNA both under the standard and anthocyanin-inducing conditions (Figure 2a). Similarly, *SlMYB32*, *SlMYB76*, and *SlTRY* mRNA levels were markedly lowered after exogenous application of the *SlMYB32*-, *SlMYB76*-, and *SlTRY*-dsRNAs under the standard and anthocyanin-inducing conditions (Figure 2b–d). Importantly, exogenous plant treatment with the nonspecific *NPTII*-dsRNA did not considerably affect the expression levels of the *SlMYBATV*, *SlMYB32*, *SlMYB76*, and *SlTRY* genes compared to the water-treated control, indicating sequence-specificity of the dsRNA-induced gene silencing effects. We also noted that when plants were grown under the anthocyanin-inducing conditions, the expression of the *SlMYBATV* gene was considerably higher both in water- and dsRNA-treated plants in comparison with plants grown under standard conditions (Figure 2a). mRNA levels of *SlMYB32, SlMYB76,* and *SlTRY* genes were reduced or remained at the same level under the anthocyanin-inducing conditions in comparison with control cultivation conditions (Figure 2b–d).

### 2.2. Exogenous dsRNAs Upregulate mRNA Levels of Anthocyanin Biosynthesis-Related Genes and Anthocyanin Content

**Figure 3:**
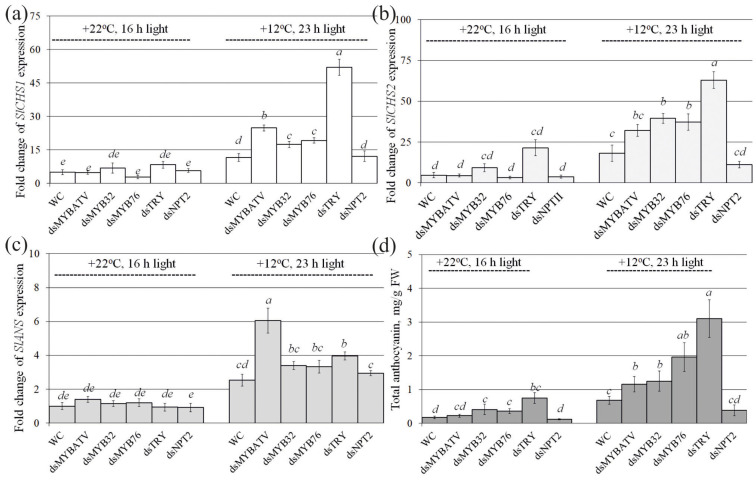
Relative fold change in *SlCHS1* (**a**), *SlCHS2* (**b**), and *SlANS* (**c**) mRNA levels after dsRNA treatments of *Solanum lycopersicum* compared to untreated plants and HPLC results (**d**) of the total anthocyanin content in the leaves of *S. lycopersicum*. WC—*S. lycopersicum* treated with sterile water; dsMYBATV—*S. lycopersicum* treated with *MYBATV*-dsRNA; dsMYB32—*S. lycopersicum* treated with *SlMYB32*-dsRNAs; dsMYB76—*S. lycopersicum* treated with *SlMYB76*-dsRNAs; dsTRY—*S. lycopersicum* treated with *SlTRY*-dsRNAs; dsNPT2—*S. lycopersicum* treated with *NPTII*-dsRNA. Total RNA was isolated seven days after dsRNA application and quantitative real-time PCR was used for gene expression analysis. *S. lycopersicum* was grown under control (+22 °C, 16 h light) and anthocyanin-inducing (+12 °C, 23 h light) conditions. Total anthocyanins are expressed as mg per g of fresh weight (mg/g FW). The data are presented as the mean ± SE (three independent experiments). Means on each figure followed by the same letter were not different using one-way analysis of variance (ANOVA), followed by the Tukey HSD multiple comparison test.

Then, we analyzed the effect of exogenous *SlMYBATV*-, *SlMYB32*-, *SlMYB76*-, *SlTRY*-, and *NPTII*-dsRNAs on the expression of three genes, encoding chalcone synthase (CHS) and anthocyanin synthase (ANS), the key enzymes involved in the biosynthesis of anthocyanins, and anthocyanin content itself in comparison with the control water treatment (Figure 3). CHS is the key enzyme in the first committed step of the biosynthesis of flavonoids, including anthocyanins, and catalyzes the stepwise condensation of 4-coumaroyl-CoA and malonyl-CoA to naringenin chalcone, while ANS catalyzes the penultimate step in the biosynthesis of the anthocyanin class of flavonoids [52].

We analyzed the expression of *SlCHS1* and *SlCHS2* genes encoding chalcone synthase and expression of the *SlANS* gene encoding anthocyanin synthase (Figure 3a–c). The analysis revealed a pronounced upregulation of *SlCHS1*, *SlCHS2*, and *SlANS* expression in *S. lycopersicum* grown under the anthocyanin-inducing conditions in comparison with the control conditions (Figure 3a–c). Under the anthocyanin-inducing conditions, expression of the *SlCHS1* gene was considerably higher in plants treated with the *SlMYBATV*-, *SlMYB32*-, *SlMYB76*-, and *SlTRY*-dsRNAs than in the water-treated and *NPTII*-dsRNA-treated *S. lycopersicum* (Figure 3a). Expression of the *SlCHS2* gene was considerably higher after application of only *SlMYB32*-, *SlMYB76*-, and *SlTRY*-dsRNAs, while expression of the *SlANS* gene was considerably higher after application of *SlMYBATV*- and *SlTRY*-dsRNAs. According to HPLC-MS analysis, this dsRNA-induced decrease in *SlMYBATV*, *SlMYB32*, *SlMYB76*, and *SlTRY* gene expression correlated with a considerable increase in anthocyanin accumulation, reaching 1.2–3.1 mg/g FW (Figure 3d). The highest anthocyanin level of 3.1 mg/g FW was detected after the application of *SlTRY*-dsRNA, which was also associated with the most pronounced increase in the expression of the *SlCHS1* and *SlCHS2* genes (Figure 3a,b,d). The application of *SlTRY*-dsRNA led to an increase in the total anthocyanin content of 4.3 times under control conditions and 4.6 times under anthocyanin-inducing conditions in comparison with the water control plants (Figure 3d). Notably, the control nonspecific *NPTII*-dsRNA did not cause any considerable alterations in both the anthocyanin content and expression of the anthocyanin biosynthesis-related genes supporting the gene-specific dsRNA-induced gene silencing effects (Figure 3a–d). 

Using HPLC with high-resolution mass spectrometry (HPLC-MS), we detected eight anthocyanin compounds in the water- and dsRNA-treated leaves of *S. lycopersicum* (Table S1). The main anthocyanins in our samples were petunidin-3-(caffeoyl)-rutinoside-5-glucoside and delphinidin-3-O-(6″-O-p-coumaroyl)-glucoside, whose contents constituted 65.9–77.9% of all individual anthocyanins (Table S1). It is possible that other anthocyanins were present in the analyzed tissues of *S. lycopersicum* but in trace amounts. Additionally, the content of most individual anthocyanins was higher in the plants grown under the anthocyanin-inducing conditions than at +22 °C (Table S1). We have shown that the treatment of plants with exogenous dsRNAs encoding the analyzed MYB-family transcription factors led to an increase in the total content of anthocyanins, mostly due to an increase in the content of petunidin-3,5-O-diglucoside, petunidin-3-(caffeoyl)-rutinoside-5-glucoside, delphinidin-3-O-(6″-O-p-coumaroyl)-glucoside and malvidin-3-(p-coumaroyl)-rutinoside-5-glucoside in the leaves of *S. lycopersicum* (Table S1).

## 3. Discussion

The growing human population and negative impact of environmental stresses promote the development of new molecular tools for crop improvement and plant protection without modification of the plant genome. Public debates about the safety of genetic engineering/genome editing technologies and current legislative limitations on the cultivation of genetically modified plants restrain the development and implementation of these tools. The development of innovative and safe approaches to improve crop traits without genome modifications is an urgent task. SIGS is currently considered as a new promising tool for plant improvement without genetic modifications and includes exogenous treatment of plant surfaces with dsRNAs or siRNAs to induce RNAi-mediated gene silencing and modify plants’ properties [13,14,27,28]. However, there is a limited number of investigations reporting on efficient gene silencing in the plant genome after external dsRNA application.

In this study, we externally applied dsRNAs to tomato foliar surfaces to silence the expression of four blockers of anthocyanin biosynthesis and to affect anthocyanin accumulation in *S. lycopersicum*, which is an important agricultural crop. While anthocyanins are produced by many plant species, they are produced at low levels or are absent in most cultivated tomatoes due to the incomplete activation of the flavonoid biosynthetic pathway [43,44]. Using the SIGS approach and qRT–PCR, we demonstrated that foliar application of dsRNAs encoding four putative transcription repressors of anthocyanin biosynthesis, SlMYBATV, SlMYB32, SlMYB76, and SlTRY, resulted in highly downregulated mRNA levels of these *S. lycopersicum* target genes and upregulated mRNA levels of anthocyanin biosynthesis genes. HPLC-MS analysis revealed that these effects were accompanied by considerably increased levels of anthocyanins. Importantly, treatment of *S. lycopersicum* with the nonspecific *NPTII*-dsRNAs had no effect on both anthocyanin production or the expression of *SlMYBATV*, *SlMYB32*, *SlMYB76*, and *SlTRY* genes of *S. lycopersicum*. This indicates that the observed dsRNA-induced MYB-family gene silencing effect was sequence-specific and was not a result of the dsRNA application itself. Several previous studies have provided evidence for the role of SlMYBATV as a negative regulator of anthocyanin biosynthesis [46,47,48], while much less is known about the role of SlMYB32, SlMYB76, and SlTRY transcription factors in the biosynthesis of anthocyanins. The available studies identified SlMYBATV, SlMYB32, SlMYB76, and SlTRY as putative MYB regulators playing a negative role in anthocyanin biosynthesis based on genome screening [46,48] or *SlTRY* gene overexpression [49]. These reports are consistent with our finding that SlMYBATV, SlMYB32, SlMYB76, and SlTRY, the four analyzed MYB regulators, act as negative regulators of anthocyanin biosynthesis.

This study revealed the total anthocyanins at the level of 0.17 mg/g FW in tomato leaves under control conditions; these results are comparable with other studies [53] reporting anthocyanin content of 0.14 mg/g FW in different tomato genotypes. Exogenous application of the MYB-specific dsRNAs in our study allowed us to increase anthocyanin levels in tomato leaves to 1.2–3.1 mg/g FW, which is a considerable elevation. 

Several tomato cultivars possessing high anthocyanin contents in their fruits and vegetative tissues have been reported and some purple fruited tomato varieties have been generated by conventional breeding [54], overexpressing selected transcription activators in transgenic tomato [55], plant microRNA858 blockage by genetic engineering [56], or CRISPR/Cas and TALEN genome editing technologies [57,58]. However, elevation of anthocyanin levels in vegetative tissues by external plant treatments is also of high interest, since anthocyanins are known to mediate stress tolerance in plants both to abiotic and biotic stresses via plant protection from growth inhibition by reducing oxidative stress, ROS scavenging, and maintaining osmotic balance [42,59]. Anthocyanin-reach tomato cultivars are known to display enhanced stress tolerance to various stress cues [60]. On the other hand, spraying plants with dsRNA solutions with the purpose of changing certain plant traits is a new tool that still needs to be developed. Here, we are still at the very beginning of the path, so changing the properties of plants in any tissues is interesting and relevant.

At present, there is a high number of studies reporting successful external dsRNA application to the plant surfaces for RNAi-mediated silencing of the target genes in infecting plant pathogens, i.e., viruses or fungi, leading to plant pathogen protection (reviewed in [8,9,14]). With regard to SIGS in the plant genome, a limited number of studies demonstrated the possibility of regulating the expression of transgenes in Arabidopsis or tobacco by transgene-specific exogenous dsRNAs [16,50,51,61,62]. There is also a low number of investigations reporting on the silencing of plant endogenous genes after exogenous dsRNA or siRNA application [30,31,32,33,34]. There were also several studies where accessory techniques, such as nanoparticles [35,36] or laser light [37], have been used to achieve efficient exogenously induced gene silencing. We developed our study based on the initial report by Numata et al. [61] who infiltrated *A. thaliana* leaves with a carrier peptide in a complex with siRNA encoding the *AtCHS* gene, and on our recent study where we demonstrated that foliar dsRNA treatments of *A. thaliana* significantly reduced the expression of the *AtCHS* gene and two genes encoding transcriptional repressors of anthocyanin biosynthesis in Arabidopsis, *AtMYBL2* and *AtANAC032* [19]. Numata et al. [61] reported a local loss of anthocyanin pigmentation by visual observation, but *AtCHS* mRNA and anthocyanin levels were not analyzed. The present study demonstrates that the foliar application of naked dsRNA (i.e., pure dsRNA, without nanoclay or other accessory components) to the leaves of *S. lycopersicum* induced the silencing of *SlMYBATV*, *SlMYB32*, *SlMYB76*, and *SlTRY* genes which was accompanied by enhanced anthocyanin accumulation, supporting the role of the four analyzed MYB regulators as negative regulators of anthocyanin biosynthesis. The capabilities of this SIGS method make it a promising one to change various characteristics of plants at different intervals of time, which is a unique feature of a biotechnological tool and opens up a great potential of this approach for use in agriculture.

In summary, in this study, we demonstrated for the first time that exogenous foliar treatments of tomato with MYB-specific dsRNAs can be used as a rapid silencing tool in plant gene functional studies to verify plant gene function, and in plant biotechnology to elevate the content of biologically active compounds.

## 4. Materials and Methods

### 4.1. Plant Material and Growth Conditions

Seeds of wild-type tomato (*S. lycopersicum* L.) cultivar Micro-Tom were used in the experiments (seed material from Laboratory of Biotechnology, Federal Scientific Center of the East Asia Terrestrial Biodiversity, Vladivostok, Russia). The tomato seeds were vaporphase sterilized as described [63] (and planted to individual pots (9 cm × 9 cm) containing 200 g of commercially available rich soil (the soil was well-irrigated by filtered water applied at the bottom of the pots). Plants were grown in a chamber (Sanyo MLR-352, Panasonic, Osaka, Japan) at a light intensity of ~120 µmol m^−2^ s^−1^ over a 16 h daily light period at 22 °C for four weeks before dsRNA treatments. After the dsRNA treatments, the *S. lycopersicum* was incubated for seven additional days either under control (+22 °C, 16 h daily light period) or anthocyanin-inducing (+12 °C, 23 h daily light period) conditions in a growth chamber (KS-200, Smolenskoye SKTB SPU, Smolensk, Russia) without further irrigation to induce anthocyanin accumulation. 

### 4.2. Isolation and Sequencing of SlMYBATV, SlMYB32, SlMYB76, and SlTRY Transcripts

Full-length coding cDNA sequences of *SlMYBATV-X1* (Solyc07g052490, 519 bp), *SlMYB32* (Solyc10g055410.1.1, 822 bp), *SlMYB76* (Solyc05g008250, 537 bp), and *SlTRY* (Solyc01g095640, 285 bp) were amplified by RT–PCR using RNA samples extracted from the adult leaves of *S. lycopersicum*. Sequence data of *SlMYBATV*, *SlMYB32*, *SlMYB76*, and *SlTRY* used in the present work for primer design can be also found in the GenBank libraries under the following accession numbers: *SlMYBATV-X1* (MF197515 or NM_001365378), *SlTRY* (XM_010328616), *SlMYB32* (NM_001247046.1), and *SlMYB76* (MF197513). The primers are listed in Table S2. The RT–PCRs were performed in a Bis-M1105 Thermal Cycler (Bis-N, Novosibirsk, Russia). The RT–PCR products were subcloned into pJET1.2/blunt and sequenced as described previously [64]. The whole genomic sequences of *S. lycopersicum* are available at the Sol Genomics Network (SGN, https://solgenomics.net/, accessed on 1 March 2023).

### 4.3. dsRNA Synthesis and Application

All dsRNAs were synthesized using the T7 RiboMAX™Express RNAi System (Promega, Madison, WI, USA). For this purpose, the cloned full-length cDNAs of *SlMYBATV*, *SlTRY*, *SlMYB32*, and *SlMYB76* were amplified by PCR for in vitro transcription and dsRNA production. The T7 promoter sequence was introduced into both the 5′ and 3′ ends of the amplified *SlMYBATV*, *SlTRY*, *SlMYB32*, and *SlMYB76* in a single PCR for each gene using primers listed in Table S2. The PCRs were performed in the Bis-M1105 Thermal Cycler programmed according to T7 RiboMAX™ Express RNAi System instructions. Then, the obtained PCR products were used as templates for in vitro transcription and dsRNA synthesis following the manufacturer’s protocol. The resultant dsRNAs were analyzed by gel electrophoresis and spectrophotometry to estimate dsRNA purity, integrity, and amount. 

The *SlMYBATV*-, *SlTRY*-, *SlMYB32*-, and *SlMYB76*-dsRNAs were applied to individual four-week-old plants of *S. lycopersicum* by spraying with 2 mL atomizer polypropylene vials. For each dsRNA treatment, 70 µg of the dsRNA was diluted in 400 µL of nuclease-free water and applied to the foliar surface (all leaves of the tomato for each type of condition were treated on both the adaxial and abaxial sides). In an independent experiment, two plants of *S. lycopersicum* were used for each type of treatment (Figure 2), i.e., two plants were treated with sterile filtered water (400 µL per plant) and two plants were treated with the dsRNA of each type (400 µL of *SlMYBATV*-, *SlTRY*-, *SlMYB32*-, and *SlMYB76*-dsRNAs per plant). Then, we divided the treated *S. lycopersicum* plants into two groups for incubation, under control conditions (+22 °C, 16 h light) and anthocyanin-inducing conditions (+12 °C, and 23 h light), for seven days (Figure 2). At least three independent experiments were performed for each type of analysis. In all experiments, the dsRNAs were applied to four-week-old plants of *S. lycopersicum* at a late day time (21:00–21:30) under low soil moisture conditions, since the conditions at the time of dsRNA application (appropriate plant age, late day time, and low soil moisture) were important parameters for successful gene silencing in *A. thaliana* according to our recent analysis [19,50,51]. Soil water content before dsRNA treatment was 50–60%.

### 4.4. RNA Isolation and Reverse Transcription

For RNA isolation, a typical adult leaf of *S. lycopersicum* was collected from an individual plant (1) before dsRNA or water application and (2) seven days post-application in an independent experiment. Total RNA was isolated using the cetyltrimethylammonium bromide (CTAB)-based protocol [65]. Complementary DNAs were synthesized using 2.5 µg of total RNA by the MMLV RT Kit (Evrogen, Moscow, Russia). The reactions were performed in 50 µL aliquots of the reaction mixture, which contained the first strand buffer, 5 µL of dNTP mix (10 mM each), 1.85 µL of oligo-(dT)15 primer (100 µM), and 4.3 µL of MMLV reverse transcriptase (100 u/µL), at 37 °C for 1.5 h. The 1 µL samples of reverse transcription products were then amplified by PCR and verified on the absence for DNA contamination using primers listed in Table S2.

### 4.5. Gene Expression Analysis by qRT–PCR

The qRT–PCRs were performed with SYBR Green I Real-time PCR dye and a real-time PCR kit (Evrogen, Moscow, Russia) as described [66] using two internal controls (*SlActin* and *SlUBI*). The expression was calculated by the 2^−ΔΔCT^ method [67]. Then, the obtained data for *MYB* expression seven days post-treatment were divided to the *MYB* expression before treatment (fold change in *MYB* expression relative to respective data before treatment). All gene identification numbers and used primers are listed in Table S2.

### 4.6. Quantification of Anthocyanins

For HPLC-MS analysis, 200 mg of treated *S. lycopersicum* leaves were frozen at −20 °C and subsequently homogenized using a mortar and a pestle. Shredded tissue was weighed and extracted for 1 d at 4 °C in 2 mL of 1% (*v*/*v*) hydrochloric acid in methanol. Then, the mixture was centrifuged at 13,500 rpm for 15 min, and 1 mL of the supernatant was transferred into another glass tube. The samples were filtered through a 0.25-um nylon membrane for further analysis. The identification of all anthocyanins was performed using a 1260 Infinity analytical HPLC system (Agilent Technologies, Santa Clara, CA, USA) coupled to a Bruker HCT ultra PTM Discovery System (Bruker Daltonik GmbH, Bremen, Germany) equipped with an electrospray ionization (ESI) source. The data for anthocyanins were acquired in a positive ion mode under the operating conditions as described [68]. The MS spectra were recorded across an *m*/*z* range of 100–1500, and the individual anthocyanins were identified as described [69]. HPLC with diode array detection (HPLC–DAD) for the quantification of all anthocyanins was performed using a HPLC LC-20AD XR analytical system (Shimadzu, Kyoto, Japan). DAD data were recorded in the 200–800 nm range, and the chromatograms for quantification were acquired at 530 nm. The chromatographic separation was performed on Shim-pack GIST C18 column (150 mm, 2.1 nm i.d., 3-_m part size; Shimadzu, Japan). Anthocyanins were separated using 0.1% formic acid and acetonitrile as mobile phases A and B, respectively, with the following elution profile: 0 to 35 min 0% of B; 35 to 40 min 40% of B; 40 to 50 min 50% of B; 50 to 65 min 100% of B. A volume of 5 µL of the sample extract was injected with a constant column temperature maintained at 40 °C and a flow rate maintained at 0.2 mL/min. All solvents were of HPLC grade. The contents of anthocyanins were determined by external standard methods using the four-point regression calibration curves built with the available standards. The commercial standard cyanidin chloride, petunidin chloride, delphinidin chloride, and malvidin chloride were obtained from Sigma-Aldrich (St. Louis, MO, USA) and used as the control.

### 4.7. Statistical Analysis

The data are presented as mean ± standard error (SE) and were tested by a paired Student’s *t*-test. The *p* < 0.05 level was selected as the point of minimal statistical significance in all analyses. At least three independent experiments were performed for each type of analysis.

## Data Availability

The data presented in this study are available within the article and Supplementary Materials.

## Supplementary Materials

The following supporting information can be downloaded at: https://www.mdpi.com/article/10.3390/ijms24119344/s1.

## References

[1] Borges F., Martienssen R.A. (2015). The expanding world of small RNAs in plants. Nat. Rev. Mol. Cell Biol..

[2] Zhao J.H., Guo H.S. (2022). RNA silencing: From discovery and elucidation to application and perspectives. J. Integr. Plant Biol..

[3] Wilson R.C., Doudna J.A. (2013). Molecular mechanisms of RNA interference. Annu. Rev. Biophys..

[4] Guleria P., Mahajan M., Bhardwaj J., Yadav S.K. (2011). Plant small RNAs: Biogenesis, mode of action and their roles in abiotic stresses. Genom. Proteom. Bioinform..

[5] Muhammad T., Zhang F., Zhang Y., Liang Y. (2019). RNA interference: A natural immune system of plants to counteract biotic stressors. Cells.

[6] Tiwari M., Sharma D., Trivedi P.K. (2014). Artificial microRNA mediated gene silencing in plants: Progress and perspectives. Plant Mol. Biol..

[7] Kamthan A., Chaudhuri A., Kamthan M., Datta A. (2015). Small RNAs in plants: Recent development and application for crop improvement. Front. Plant Sci..

[8] Morozov S.Y., Solovyev A.G., Kalinina N.O., Taliansky M.E. (2019). Double-stranded RNAs in plant protection against pathogenic organisms and viruses in agriculture. Acta Nat..

[9] Gebremichael D.E., Haile Z.M., Negrini F., Sabbadini S., Capriotti L., Mezzetti B., Baraldi E. (2021). RNA interference strategies for future management of plant pathogenic fungi: Prospects and challenges. Plants.

[10] Koch A., Wassenegger M. (2021). Host-induced gene silencing-mechanisms and applications. New Phytol..

[11] Ramegowda V., Mysore K.S., Senthil-Kumar M. (2014). Virus-induced gene silencing is a versatile tool for unraveling the functional relevance of multiple abiotic-stress-responsive genes in crop plants. Front. Plant Sci..

[12] Mamta B., Rajam M.V. (2017). RNAi technology: A new platform for crop pest control. Physiol. Mol. Biol. Plants.

[13] Hoang B.T.L., Fletcher S.J., Brosnan C.A., Ghodke A.B., Manzie N., Mitter N. (2022). RNAi as a foliar spray: Efficiency and challenges to field applications. Int. J. Mol. Sci..

[14] Dubrovina A.S., Kiselev K.V. (2019). Exogenous RNAs for gene regulation and plant resistance. Int. J. Mol. Sci..

[15] Dalakouras A., Wassenegger M., McMillan J.N., Cardoza V., Maegele I., Dadami E., Runne M., Krczal G., Wassenegger M. (2016). Induction of silencing in plants by high-pressure spraying of *in vitro*-synthesized small RNAs. Front. Plant Sci..

[16] Mitter N., Worrall E.A., Robinson K.E., Li P., Jain R.G., Taochy C., Fletcher S.J., Carroll B.J., Lu G.Q., Xu Z.P. (2017). Clay nanosheets for topical delivery of RNAi for sustained protection against plant viruses. Nat. Plants.

[17] Koch A., Biedenkopf D., Furch A., Weber L., Rossbach O., Abdellatef E., Linicus L., Johannsmeier J., Jelonek L., Goesmann A. (2016). An RNAi-based control of *Fusarium graminearum* infections through spraying of long dsRNAs involves a plant passage and is controlled by the fungal silencing machinery. PLoS Pathog..

[18] Pampolini F., Rodrigues T.B., Leelesh R.S., Kawashima T., Rieske L.K. (2020). Confocal microscopy provides visual evidence and confirms the feasibility of dsRNA delivery to emerald ash borer through plant tissues. J. Pest Sci..

[19] Kiselev K.V., Suprun A.R., Aleynova O.A., Ogneva Z.V., Kalachev A.V., Dubrovina A.S. (2021). External dsRNA downregulates anthocyanin biosynthesis-related genes and affects anthocyanin accumulation in *Arabidopsis thaliana*. Int. J. Mol. Sci..

[20] Konakalla N.C., Kaldis A., Berbati M., Masarapu H., Voloudakis A.E. (2016). Exogenous application of double-stranded RNA molecules from TMV *p126* and *CP* genes confers resistance against TMV in tobacco. Planta.

[21] Song X.S., Gu K.X., Duan X.X., Xiao X.M., Hou Y.P., Duan Y.B., Wang J.X., Zhou M.G. (2018). A myosin5 dsRNA that reduces the fungicide resistance and pathogenicity of *Fusarium asiaticum*. Pest. Biochem. Physiol..

[22] Song X.S., Gu K.X., Duan X.X., Xiao X.M., Hou Y.P., Duan Y.B., Wang J.X., Yu N., Zhou M.G. (2018). Secondary amplification of siRNA machinery limits the application of spray-induced gene silencing. Mol. Plant Pathol..

[23] Kaldis A., Berbati M., Melita O., Reppa C., Holeva M., Otten P., Voloudakis A. (2008). Exogenously applied dsRNA molecules deriving from the Zucchini yellow mosaic virus (ZYMV) genome move systemically and protect cucurbits against ZYMV. Mol. Plant Pathol..

[24] Gu K.X., Song X.S., Xiao X.M., Duan X.X., Wang J.X., Duan Y.B., Hou Y.P., Zhou M.G. (2019). A β2-tubulin dsRNA derived from *Fusarium asiaticum* confers plant resistance to multiple phytopathogens and reduces fungicide resistance. Pest. Biochem. Physiol..

[25] Werner B.T., Gaffar F.Y., Schuemann J., Biedenkopf D., Koch A.M. (2020). RNA-spray-mediated silencing of *Fusarium graminearum AGO* and *DCL* genes improve barley disease resistance. Front. Plant Sci..

[26] Qiao L., Lan C., Capriotti L., Ah-Fong A., Nino Sanchez J., Hamby R., Heller J., Zhao H., Glass N.L., Judelson H.S. (2021). Spray-induced gene silencing for disease control is dependent on the efficiency of pathogen RNA uptake. Plant Biotechnol. J..

[27] Wang M., Jin H. (2017). Spray-induced gene silencing: A powerful innovative strategy for crop protection. Trends Microbiol..

[28] Das P.R., Sherif S.M. (2020). Application of exogenous dsRNAs-induced RNAi in agriculture: Challenges and triumphs. Front. Plant Sci..

[29] Akbar S., Wei Y., Zhang M.-Q. (2022). RNA interference: Promising approach to combat plant viruses. Int. J. Mol. Sci..

[30] Lau S.E., Schwarzacher T., Othman R.Y., Harikrishna J.A. (2015). dsRNA silencing of an R2R3-MYB transcription factor affects flower cell shape in a Dendrobium hybrid. BMC Plant Biol..

[31] Li H., Guan R., Guo H., Miao X. (2015). New insights into an RNAi approach for plant defence against piercing-sucking and stem-borer insect pests. Plant Cell Environ..

[32] Marcianò D., Ricciardi V., Fassolo E.M., Passera A., BIANCO P.A., Failla O., Casati P., Maddalena G., De Lorenzis G., Toffolatti S.L. (2021). RNAi of a putative grapevine susceptibility gene as a possible downy mildew control strategy. Front. Plant Sci..

[33] Nerva L., Guaschino M., Pagliarani C., De Rosso M., Lovisolo C., Chitarra W. (2022). Spray-induced gene silencing targeting a glutathione S-transferase gene improves resilience to drought in grapevine. Plant Cell Environ..

[34] Sammons R., Ivashuta S., Liu H., Wang D., Feng P., Kouranov A., Andersen S. (2015). Polynucleotide molecules for gene regulation in plants. U.S. Patent.

[35] Jiang L., Ding L., He B., Shen J., Xu Z., Yin M., Zhang X. (2014). Systemic gene silencing in plants triggered by fluorescent nanoparticle-delivered double-stranded RNA. Nanoscale.

[36] Molesini B., Pennisi F., Cressoni C., Vitulo N., Dusi V., Speghini A., Pandolfini T. (2022). Nanovector-mediated exogenous delivery of dsRNA induces silencing of target genes in very young tomato flower buds. Nanoscale Adv..

[37] Killiny N., Gonzalez-Blanco P., Gowda S., Martini X., Etxeberria E. (2021). Plant functional genomics in a few days: Laser-assisted delivery of double-stranded RNA to higher plants. Plants.

[38] Nityagovsky N.N., Kiselev K.V., Suprun A.R., Dubrovina A.S. (2022). Exogenous dsRNA induces RNA interference of a chalcone synthase gene in *Arabidopsis thaliana*. Int. J. Mol. Sci..

[39] Kong J.M., Chia L.S., Goh N.K., Chia T.F., Brouillard R. (2003). Analysis and biological activities of anthocyanins. Phytochemistry.

[40] Khoo H.E., Azlan A., Tang S.T., Lim S.M. (2017). Anthocyanidins and anthocyanins: Colored pigments as food, pharmaceutical ingredients, and the potential health benefits. Food Nutr. Res..

[41] Konczak I., Zhang W. (2004). Anthocyanins—More than nature’s colours. J. Biomed. Biotech..

[42] Kaur S., Tiwari V., Kumari A., Chaudhary E., Sharma A., Ali U., Garg M. (2023). Protective and defensive role of anthocyanins under plant abiotic and biotic stresses: An emerging application in sustainable agriculture. J. Biotechnol..

[43] Verhoeyen M.E., Bovy A., Collins G., Muir S., Robinson S., De Vos CH R., Colliver S. (2002). Increasing antioxidant levels in tomatoes through modification of the flavonoid biosynthetic pathway. J. Exp. Bot..

[44] Gonzali S., Mazzucato A., Perata P. (2009). Purple as a tomato: Towards high anthocyanin tomatoes. Trends Plant Sci..

[45] Chaves-Silva S., Dos Santos A.L., Chalfun-Júnior A., Zhao J., Peres L.E.P., Benedito V.A. (2018). Understanding the genetic regulation of anthocyanin biosynthesis in plants—Tools for breeding purple varieties of fruits and vegetables. Phytochemistry.

[46] Cao X., Qiu Z., Wang X., Van Giang T., Liu X., Wang J., Wang X., Gao J., Guo Y., Du Y. (2017). A putative R3 MYB repressor is the candidate gene underlying atroviolacium, a locus for anthocyanin pigmentation in tomato fruit. J. Exp Bot..

[47] Colanero S., Perata P., Gonzali S. (2018). The *atroviolacea* gene encodes an R3-MYB protein repressing anthocyanin synthesis in tomato plants. Front. Plant Sci..

[48] Zhao P., Li Q., Li J., Wang L., Ren Z. (2014). Genome-wide identification and characterization of R2R3MYB family in Solanum lycopersicum. Mol. Gen. Genom..

[49] Nukumizu Y., Wada T., Tominaga-Wada R. (2013). Tomato (*Solanum lycopersicum*) homologs of TRIPTYCHON (SlTRY) and GLABRA3 (SlGL3) are involved in anthocyanin accumulation. Plant Signal Behav..

[50] Kiselev K.V., Suprun A.R., Aleynova O.A., Ogneva Z.V., Kostetsky E.Y., Dubrovina A.S. (2022). The specificity of transgene suppression in plants by exogenous dsRNA. Plants.

[51] Kiselev K.V., Suprun A.R., Aleynova O.A., Ogneva Z.V., Dubrovina A.S. (2021). Physiological conditions and dsRNA application approaches for exogenously induced RNA interference in *Arabidopsis thaliana*. Plants.

[52] Liu Y., Tikunov Y., Schouten R.E., Marcelis L.F.M., Visser R.G.F., Bovy A. (2018). Anthocyanin biosynthesis and degradation mechanisms in solanaceous vegetables: A review. Front Chem..

[53] Tudor-Radu M., Vijan L.E., Tudor-Radu C.M., Tita I., Sima R., Mitrea R. (2016). Assessment of Ascorbic Acid, Polyphenols, Flavonoids, Anthocyanins and Carotenoids Content in Tomato Fruits. Not. Bot. Horti Agrobot. Cluj-Napoca.

[54] Mazzucato A., Willems D., Bernini R., Picarella M.E., Santangelo E., Ruiu F., Tilesi F., Soressi G.P. (2013). Novel phenotypes related to the breeding of purple-fruited tomatoes and effect of peel extracts on human cancer cell proliferation. Plant Physiol. Biochem..

[55] Butelli E., Titta L., Giorgio M., Mock H.P., Matros A., Peterek S., Schijlen E.G., Hall R.D., Bovy A.G., Luo J. (2008). Enrichment of tomato fruit with health-promoting anthocyanins by expression of select transcription factors. Nat. Biotechnol..

[56] Jia X., Shen J., Liu H., Li F., Ding N., Gao C., Pattanaik S., Patra B., Li R., Yuan L. (2015). Small tandem target mimic-mediated blockage of microRNA858 induces anthocyanin accumulation in tomato. Planta.

[57] Čermák T., Baltes N.J., Čegan R., Zhang Y., Voytas D.F. (2015). High-frequency, precise modification of the tomato genome. Genome Biol..

[58] Tiwari J.K., Singh A.K., Behera T.K. (2023). CRISPR/Cas genome editing in tomato improvement: Advances and applications. Front Plant Sci..

[59] Naing A.H., Kim C.K. (2021). Abiotic stress-induced anthocyanins in plants: Their role in tolerance to abiotic stresses. Physiol. Plant..

[60] Faqir Napar W.P., Kaleri A.R., Ahmed A., Nabi F., Sajid S., Ćosić T., Yao Y., Liu J., Raspor M., Gao Y. (2022). The anthocyanin-rich tomato genotype LA-1996 displays superior efficiency of mechanisms of tolerance to salinity and drought. J. Plant Physiol..

[61] Numata K., Ohtani M., Yoshizumi T., Demura T., Kodama Y. (2014). Local gene silencing in plants via synthetic dsRNA and carrier peptide. Plant Biotechnol. J..

[62] Dubrovina A.S., Aleynova O.A., Kalachev A.V., Suprun A.R., Ogneva Z.V., Kiselev K.V. (2019). Induction of transgene suppression in plants via external application of synthetic dsRNA. Int. J. Mol. Sci..

[63] Dubrovina A.S., Aleynova O.A., Suprun A.R., Ogneva Z.V., Kiselev K.V. (2020). Transgene suppression in plants by foliar application of *in vitro*-synthesized small interfering RNAs. Appl. Microbiol. Biotechnol..

[64] Dubrovina A.S., Aleynova O.A., Ogneva Z.V., Suprun A.R., Ananev A.A., Kiselev K.V. (2019). The effect of abiotic stress conditions on expression of calmodulin (*CaM*) and calmodulin-like (*CML*) genes in wild-growing grapevine *Vitis amurensis*. Plants.

[65] Kiselev K.V., Dubrovina A.S., Shumakova O.A., Karetin Y.A., Manyakhin A.Y. (2013). Structure and expression profiling of a novel calcium-dependent protein kinase gene, *CDPK3a*, in leaves, stems, grapes, and cell cultures of wild-growing grapevine *Vitis amurensis* Rupr. Plant Cell Rep..

[66] Dubrovina A.S., Kiselev K.V. (2019). The role of calcium-dependent protein kinase genes *VaCPK1* and *VaCPK26* in the response of *Vitis amurensis* (in vitro) and *Arabidopsis thaliana* (in vivo) to abiotic stresses. Russ. J. Genet..

[67] Livak K.J., Schmittgen T.D. (2001). Analysis of relative gene expression data using real-time quantitative PCR and the 2(-Delta Delta C(T)) method. Methods.

[68] Abdullin S.R., Nikulin V.Y., Nikulin A.Y., Manyakhin A.Y., Bagmet V.B., Suprun A.R., Gontcharov A.A. (2021). *Roholtiella mixta* sp. nov. (Nostocales, Cyanobacteria): Morphology, molecular phylogeny, and carotenoid content. Phycologia.

[69] Wang H., Sun S., Zhou Z., Qiu Z., Cui X. (2020). Rapid analysis of anthocyanin and its structural modifications in fresh tomato fruit. Food Chem..

