# Supplementary material for: Exogenously Induced Silencing of Four MYB Transcription Repressor Genes and Activation of Anthocyanin Accumulation in Solanum lycopersicum
Source: Int J Mol Sci. 2023 May 26;24(11):9344. doi: 10.3390/ijms24119344 (PMC10253785; doi:10.3390/ijms24119344)
Supplement: Supplementary file 1 [file ijms-24-09344-s001.zip › ijms-2397449-supplementary/Supplementary Table S1.pdf]

Supplementary Table S1. The content of anthocyanins in mg per g of fresh weight (mg/g FW) in the leaves of *Solanum lycopersicum* grown under the control (+22 °C, 16 h light) and anthocyanin-inducing (+12 °C, 23 h light) conditions. WC – *S. lycopersicum* treated with sterile water, MYB1— *S. lycopersicum* treated with *SlMYBL1*-dsRNA; TRY— *S. lycopersicum* treated with *SlTRY*-dsRNA; MYB76— *S. lycopersicum* treated with *SlMYB76*-dsRNA; MYB32 — *S. lycopersicum* treated with *SlMYB32*-dsRNA; NPT2— *S. lycopersicum* treated with *NPTII*-dsRNA. The data are presented as the mean ± SE (three independent experiments). Means followed by the same letter in one row were not different using Student's t test. $p < 0.05$ was considered to be statistically significant.

Compounds	22 WC	22 MYB1	22 TRY	22 MYB76	22 MYB32	22 NPTII	12 WC	12 MYB1	12 TRY	12 MYB76	12 MYB32	12 NPTII
Petunidin-3,5-O-diglucoside	0.014± 0.006^{f}	0.026± 0.011^{f}	0.120± 0.027^{bc}	0.060± 0.022^{de}	0.041± 0.026^{ef}	0.017± 0.01^{f}	0.066± 0.011^{de}	0.091± 0.018^{cd}	0.246± 0.033^{a}	0.170± 0.029^{b}	0.109± 0.018^{bc}	0.021± 0.009^{f}
Petunidin-3-(caffeoyl)-rutinoside-5-glucoside	0.039± 0.012^{ef}	0.046± 0.011^{ef}	0.056± 0.014^{de}	0.061± 0.004^{de}	0.073± 0.008^{de}	0.028± 0.016^{f}	0.131± 0.025^{cd}	0.242± 0.054^{bc}	0.611± 0.121^{a}	0.371± 0.090^{ab}	0.252± 0.078^{bc}	0.082± 0.060^{def}
Petunidin-3-(p-coumaroyl)-rutinoside-5-glucoside	0.002± 0.001^{bc}	0 ^{c}	0.004± 0.003^{bc}	0^{c}	0.006± 0.003^{ab}	0^{c}	0.003± 0.001^{bc}	0.023± 0.011^{a}	0.013± 0.007^{ab}	0^{c}	0.004± 0.003^{bc}	0^{c}
Petunidin-3-(feruloyll)-rutinoside-5-glucoside	0.009± 0.008^{abc}	0.003± 0.001^{abc}	0.007± 0.003^{ab}	0.003± 0.002^{abc}	0^{c}	0^{c}	0.056± 0.054^{ab}	0.011± 0.005^{ab}	0.021± 0.010^{ab}	0.017± 0.006^{ab}	0.008± 0.004 ^{ab}	0.007± 0.006^{abc}
Delphinidin-3-O-(6"-O-p-coumaroyl)-glucoside	0.079± 0.019^{g}	0.103± 0.022^{fg}	0.434± 0.095^{de}	0.163± 0.034^{ef}	0.233± 0.103^{ef}	0.044± 0.007^{g}	0.324± 0.058^{de}	0.575± 0.125^{cd}	1.809± 0.343^{a}	1.106± 0.24^{b}	0.704± 0.195^{bc}	0.176± 0.067^{ef}
Delphinidin-3-O-glucoside	0.018± 0.003^{f}	0.028± 0.004^{ef}	0.054± 0.013^{de}	0.049± 0.009^{de}	0.030± 0.005^{ef}	0.022± 0.002^{f}	0.055± 0.007^{de}	0.125± 0.021^{bc}	0.185± 0.024^{a}	0.154± 0.024^{ab}	0.099± 0.006^{cd}	0.059± 0.018^{de}
Malvidin-3-(p-coumaroyl)-rutinoside-5-glucoside	0.010± 0.003^{ef}	0.012± 0.005^{ef}	0.060± 0.018^{cd}	0.015± 0.004^{ef}	0.014± 0.011^{ef}	0.003± 0.002^{f}	0.037± 0.009^{de}	0.064± 0.017^{bc}	0.180± 0.037^{a}	0.114± 0.027^{ab}	0.060± 0.012^{cd}	0.021± 0.011^{de}
Cyanidin-3-O-(6"-O-p-coumaroyl)-glucoside	0.003± 0.001^{cd}	0.008± 0.002^{bc}	0.016± 0.006^{bc}	0.010± 0.003^{bc}	0.012± 0.006^{bc}	0^{d}	0.009± 0.002^{bc}	0.026± 0.004^{ab}	0.039± 0.005^{a}	0.030± 0.006^{ab}	0.015± 0.005^{bc}	0.016± 0.006^{bc}
