# Supplementary material for: Exogenously Induced Silencing of Four MYB Transcription Repressor Genes and Activation of Anthocyanin Accumulation in Solanum lycopersicum
Source: Int J Mol Sci. 2023 May 26;24(11):9344. doi: 10.3390/ijms24119344 (PMC10253785; doi:10.3390/ijms24119344)
Supplement: Supplementary file 1 [file ijms-24-09344-s001.zip › ijms-2397449-supplementary/Supplementary Table S2.pdf]

Supplementary Table S1 Primers used in RT-PCR and qRT-PCRs.

Gene name (ID number)	Primer name	Primers, 5′-3′
Primers for cloning full-length cDNA coding sequences, 5′-3′		
SlMYB-ATV (Solyc07g052490)	SlMyb1-nachS1<br>SlMyb1-konA1	5′ATGGTAAGAGCTCCTTGTTG<br>5′TCAAAGCTCCTGTAAGCCGC
SlTRY (Solyc01g095640)	SlTRY-nachS1<br>SlTRY-konA1	5′ATGGATCAAAATCTCCATCA<br>5′TTATGTAGGTGGTAGACTTT
SlMYB32 (Solyc10g055410.1.1)	SlMyb32-nachS1<br>SlMyb32-konA1	5′ATGGGAAGGTCACCTTGTTG<br>5′TCACTTAGTTTCCAAAGTTC
SlMYB76 (Solyc05g008250)	SlMYB76-nachS1<br>SlMYB76-konA1	5′ATGAGAAAGCCTTGTTGTGA<br>5′CTATGGAATTAAATTGAGATC
Specific primers for dsRNA design, 5′-3′		
SlMYBATV (Solyc07g052490)	SlMyb-dsRNA-s1<br>SlMyb-dsRNA-a1	5′TAATACGACTCACTATAGGGAGAGAGCCCTTCCAA AACAAGCC<br>5′TAATACGACTCACTATAGGGAGATCAAAGCTCCTG TAAGCCGC
SlTRY (Solyc01g095640)	SlTRY-dsRNA-s1<br>SlTRY-dsRNA-a1	5′TAATACGACTCACTATAGGGAGAATGGATCAAAAT CTCCATCACCG<br>5′TAATACGACTCACTATAGGGAGATTATGTAGGTGG TAGACTTTTCTTAAT
SlMYB32 (Solyc10g055410.1.1)	SlMyb32-dsRNA-s1<br>SlMyb32-dsRNA-a1	5′TAATACGACTCACTATAGGGAGACAGGAAGATTAC CAGGAAGAACAG<br>5′TAATACGACTCACTATAGGGAGACAAGCCCAAAAA GTCATAACCAG
SlMYB76 (Solyc05g008250)	SlMYB76-dsRNA-s1<br>SlMYB76-dsRNA-a1	5′TAATACGACTCACTATAGGGAGAGTTGTAGGCTAA GATGGATGAATT<br>5′TAATACGACTCACTATAGGGAGAGAGATCAAGCAA TGAACTTGTAATTTG
NPTII (GenBank AY818371)	npt-T71-s<br>npt-T72-a	5′TAATACGACTCACTATAGGGAGAATGTGGATTGAA CAAGATGGATTG<br>5′TAATACGACTCACTATAGGGAGATCCACCATGATA TTCGGCAAGCAG
Primers for cDNA check-up on DNA contamination, 5′-3′		
SlActin (Solyc04g011500.2)	SlAct-OT-s<br>SlAct-OT-a	5′ATGGCAGACGGAGAGGAT<br>5′TTCACGATTAGCCTTTGG GT
Primers for PCR and real-time PCR, 5′-3′		
SlMYBATV (Solyc07g052490)	SlMyb1-realS1<br>SlMyb1-realA1	5′TGGACGACTCAGGAAGATCA<br>5′GAGTCTGCAACTCTTCCCAC
SlTRY (Solyc01g095640)	SlTRY-realS1<br>SlTRY-realA1	5′CAGCAACTCTCTGCTAGTCC<br>5′GGTGCATGAGTTTGTGTCGGTGATG
SlMyb32 (Solyc10g055410.1.1)	SlMyb32-realS1<br>SlMyb32-realA1	5′GGCACATACAAACAAAGGAGCATGGAC<br>5′CGGAGACGACAACTTTTACCGCATC
SlMYB76 (Solyc05g008250)	SlMYB76-realS1<br>SlMYB76-realA1	5′TGCTGGCCTGCTTCGTTGTG<br>5′CGTTCGTCCTGGCAATCTTCCTG
SlCHS1 (Solyc09g091510.2.1)	SlCHS1-reals1<br>SlCHS1-realA1	5′ACTCGTCTCAGCAGCCCAAACTC<br>5′AAGCCCAACCTCACGTAGGTGTCC
SlCHS2 (Solyc05g053550.2.1)	SlCHS2-reals1<br>SlCHS2-realA1	5′AGAAGCAGCCCAAAAGGCCATTAAAG<br>5′CGGATAACGGTCCCACCAGCAAAG
SlANS (Solyc08g080040.2)	SlANS-realS1<br>SlANS-realA1	5′TCTGGCCTAAAACCCCTGCTGAC<br>5′TCCTTCCTCCAATCCCAACCCAATC

SlActin (Solyc04g011500.2)	SlAct-realS<br>SlAct-realA	5′GAAATAGCATAAGATGGCAGACG<br>5′ATACCCACCATCACACCAGTAT
SlUBI (Solyc07g064130.1)	SlUBI-realS<br>SlUBI-realA	5′GGACGGACGTACTCTAGCTGAT<br>5′AGCTTTCGACCTCAAGGGTA
NPTII (GenBank AJ414108)	nptII-realS<br>nptII-realA	5′TTGCTGAAGAGCTTGGCGGCGAAT<br>5′TCAGAAGAACTCGTCAAGAAGG
